# Application of the perturbation iteration method to boundary layer type problems

**DOI:** 10.1186/s40064-016-1859-4

**Published:** 2016-02-29

**Authors:** Mehmet Pakdemirli

**Affiliations:** Applied Mathematics and Computation Center, Celal Bayar University, 45140 Muradiye, Manisa, Turkey

**Keywords:** Perturbation methods, Perturbation–iteration algorithm, Boundary layer problems, Ordinary differential equations, Singular perturbation problems, 34B05, 34B15, 34B16, 34E10

## Abstract

The recently developed perturbation iteration method is applied to boundary layer type singular problems for the first time. As a preliminary work on the topic, the simplest algorithm of PIA(1,1) is employed in the calculations. Linear and nonlinear problems are solved to outline the basic ideas of the new solution technique. The inner and outer solutions are determined with the iteration algorithm and matched to construct a composite expansion valid within all parts of the domain. The solutions are contrasted with the available exact or numerical solutions. It is shown that the perturbation–iteration algorithm can be effectively used for solving boundary layer type problems.

## Background

The small parameter assumption is one of the most characteristic limitations of the perturbation methods. For nonlinear problems, this corresponds to a weakly nonlinear system. In recent decades, efforts have been intensified to overcome the limitation of the perturbation methods and to produce uniform solutions for large perturbation parameters.

One such attempt is to incorporate perturbation solutions with the iteration procedures to advance to the real solutions for large parameters. For algebraic equations, the systematic way of constructing single point iteration algorithms for root finding was outlined in the pioneering work due to Pakdemirli and Boyacı (2007). It was shown that many different root finding algorithms, some of which are original can be constructed by the perturbation–iteration algorithms. The algorithms are classified as PIA(*n*,*m*), *n* representing the number of correction terms in the perturbation expansion and *m* representing the highest order of derivatives in the Taylor series expansions. Although PIA(1,1) corresponds to the well-known Newton–Raphson algorithm, many different algorithms non-existent in the literature can be constructed with different selections of the numbers *n* and *m* with *n* ≤ *m* (Pakdemirli and Boyacı 2007; Pakdemirli et al. 2007, 2008).

The classification of such algorithms for algebraic equations turns out to be quite original. Motivated by the successful results, the systematic and algorithmic way of incorporating the methods were applied to first order ordinary differential equations in the pioneering work of Pakdemirli et al. (2011). The extension of the method to differential equations is non-trivial as the dependent variable and its derivatives are considered as separate variables inspired from the Lie Group theory applied to differential equations. The method is called as “the perturbation iteration method” to distinguish it from the past literature on the so-called “iteration-perturbation methods” (He 2001; Mickens 1987, 2005, 2006) which are not systematic approaches and does not produce general algorithms valid for various types of differential equations. The new perturbation–iteration method is directly applicable in a systematic algorithmic way, does not require special transformations or *ad hoc* assumptions. Second order differential equations were treated by the same method by Aksoy and Pakdemirli (2010) for Bratu type equations. Aksoy et al. (2012) further solved some nonlinear heat transfer equations. Dolapci et al. (2013) applied the method to Fredholm and Volterra integral equations. Şenol et al. (2013) treated the first order differential equation systems using the method. Pakdemirli (2013) reviewed the mentioned work. The basic idea behind the new method is to construct an approximate solution as in the classical perturbation methods, but then to iterate over this approximate solution to converge to the real solution of the problem. The iterations indeed enable one to converge to the real solutions for large perturbation parameters also. The mentioned work contains only applications to the regular perturbation problems which do not have secular terms in the analysis. In a very recent work, Pakdemirli (2015) successfully applied the method to problems with secularities also. The work considers the modifications of the method for non-regular problems containing secular terms which possess slow convergence problems and truncation leads to unphysical unbounded solutions.

So far, the method has not been applied to boundary layer type singular problems which remain an open area of research. The work presented here is an initial attempt to address the implementation of the method to such singular problems. It is well known that for such problems, the method of matched asymptotic expansions is the most reliable method. In the method of matched asymptotic expansions, a solution valid outside the boundary layer is constructed first, which is the regular solution of the original problem, named the outer solution. Then another solution inside the boundary layer region where abrupt changes in the solutions is observed is constructed, called the inner solution. The outer and inner solutions are matched over the overlapping region and a composite expansion valid throughout the whole domain is constructed as a final step.

In the previous work, the systematic way of constructing perturbation–iteration algorithms are named as PIA(*n*,*m*), *n* representing the number of correction terms in the perturbation expansion and *m* representing the order of highest derivatives in the Taylor series expansion. The simplest algorithm is the PIA(1,1) with one correction term in the perturbation expansion and first order derivatives in the Taylor series expansion. For consistency and solvable iteration equations, *n* should be equal or less than *m* (*n* ≤ *m*). As *n* or *m* increases, the convergence became faster but the algebraic complexity of the equations may end up with analytically unsolvable equations. In the previous work on differential equations, solutions are presented for PIA(1,1), PIA(1,2), PIA(1,3) and PIA(2,2) algorithms. In this pioneering and preliminary work on boundary layers, only the PIA(1,1) algorithms are applied to boundary layer type problems. Linear and nonlinear problems are treated with the PIA(1,1) method and solutions are contrasted with the available exact or numerical solutions of the original problem. For PIA(1,1), since the order of equations to be solved is less than the number of boundary conditions, the outer and inner solutions must be found by separate iterations and matched together similar to the method of matched asymptotic expansions.

Finally, as a last comment, the Lie Group methods of constructing exact solutions to differential equations cannot be applied to the problems considered here. The main reason is that the problems are defined over finite domains. For such domains, the symmetries of the equations do not remain stable after the boundary conditions are applied. To obtain group invariant solutions (similarity solutions) the equations as well as the boundary conditions and boundaries should remain invariant under the transformations especially for nonlinear problems (Bluman and Kumei 1989). For infinite or semi-infinite domains, this is more likely to occur but for finite domains, the symmetries are lost. For the singular problems with finite domains, the perturbation solutions, especially the method of matched asymptotic expansions is one of the most reliable analytical method. Here, an alternative approach which is a modification of the perturbation–iteration method to handle such problems is developed.

## Theory of the perturbation iteration algorithm for second order differential equations

In this section, PIA(1,1) algorithm is derived for second order differential equations. In accordance, only one correction term in the perturbation expansion and Taylor expansions up to first order derivatives are taken in constructing the iteration procedure. Consider a general second order differential equation,

1 $$F(y^{\prime \prime } ,y^{\prime } ,y,\varepsilon ) = 0$$

with *y* = *y*(*x*) and *ε* is the perturbation parameter. Note that a parameter such as *ε* may not exist at all in the original equation. In that case, the parameter should carefully be inserted into the appropriate part of the equation. The role of the perturbation parameter is to simplify the resulting iteration equations which cannot be solved otherwise. If the parameter is artificially introduced, it can be taken as 1 at the end. Only one correction term is taken in the perturbation expansion

2 $$y_{n + 1} = y_{n} + \varepsilon (y_{c} )_{n}$$

Upon substitution of (2) into (1) and expanding in a Taylor series with first order derivatives only yields

3 $$\begin{aligned} & F(y_{n}^{\prime \prime } ,y_{n}^{\prime } ,y_{n} ,0) + F_{y} (y_{n}^{\prime \prime } ,y_{n}^{\prime } ,y_{n} ,0)\,\varepsilon \,(y_{c} )_{n} + F_{{y^{\prime } }} (y_{n}^{\prime \prime } ,y_{n}^{\prime } ,y_{n} ,0)\varepsilon \,(y_{c}^{\prime } )_{n} \\ & \quad + F_{{y^{\prime \prime } }} (y_{n}^{\prime \prime } ,y_{n}^{\prime } ,y_{n} ,0)\varepsilon \,(y^{\prime\prime}_{c} )_{n} + F_{\varepsilon } (y_{n}^{\prime \prime } ,y_{n}^{\prime } ,y_{n} ,0)\,\varepsilon = 0 \\ \end{aligned}$$

where $F_{y} = \frac{{\partial {\text{F}}}}{{\partial {\text{y}}}}$, $F_{{y^{\prime } }} = \frac{\partial F}{{\partial y^{\prime } }}$, $F_{{y^{\prime \prime } }} = \frac{\partial F}{{\partial y^{\prime \prime } }}$, $F_{\varepsilon } = \frac{\partial F}{\partial \varepsilon }$ and all derivatives are evaluated at *ε* = 0. It is readily observed that the above equation is a variable coefficient non-homogenous linear second order differential equation with respect to the unknown $(y_{c} )_{n}$ in its most general form. Starting with an initial guess *y*_0_, first $(y_{\text{c}} )_{0}$ is calculated from (3) and then substituted into (2) to calculate *y*_1_. The iteration procedure is repeated using (3) and (2) until a satisfactory result is obtained. In solving boundary layer problems, for outer solution, the original equation is iterated. However for constructing the inner solution, first the equation is expressed in terms of the boundary layer variable and then the iteration is performed over the transformed equation. The last step is to match the solutions and construct a composite expansion valid within all the domain. This last step is similar to the calculations of the method of matched asymptotic expansions. If the inner and outer solutions cannot be matched, possible reasons may be the wrong location of the boundary layer or a bad initial guess for the iterations.

## Applications to linear differential equations

Two linear second order constant coefficient problems, one homogenous and the other non-homogenous are treated using the PIA(1,1) algorithm.

### Example Problem 1

Consider the singular perturbation problem

4 $$\varepsilon y^{{\prime \prime }} + 2y^{{\prime }} - y = 0\quad y(0) = 0,\quad y(1) = 1$$

For the problem, an outer solution and an inner solution will be sought and both solutions will be matched to construct a composite solution.

### The outer solution

To find the outer solution, the original equation is taken in the analysis

5 $$F(y,y^{{\prime }} ,y^{{\prime \prime }} ,\varepsilon ) = \varepsilon y^{{\prime \prime }} + 2y^{{\prime }} - y = 0$$

Before starting, one of the important issues is the location of the boundary layer. This requires knowledge of the physics of the problem. For variable coefficient linear second order singular equations, a systematic way of locating the boundary condition indeed exists (Nayfeh 1981). For the problem considered, the boundary layer is located near *x* ≈ 0. Therefore, the outer solution is not expected to satisfy the condition at *x* = 0. Substituting (5) into (3) and re-arranging, the iteration equation is

6 $$2y_{n}^{\prime } - y_{n} - \varepsilon (y_{c} )_{n} + 2\varepsilon (y_{c}^{\prime } )_{n} + \varepsilon y_{n}^{\prime \prime } = 0,\quad n = 0,1,2, \ldots$$

Note that due to the small term multiplying the highest derivative, the original equation yields a first order iteration equation which leads to inconsistencies if both conditions are forced to satisfy the solution. This justifies an outer and inner solutions to be constructed separately.

Inspired by the condition at the right, a simple initial guess is suggested

7 $$y_{0} = 1$$

to start the iteration. Substituting this initial guess into (6) and solving for $(y_{c} )_{0}$ yields

8 $$(y_{c} )_{0} = - \frac{1}{\varepsilon } + \frac{{c_{1} }}{2}e^{x/2}$$

Hence, the first iteration result is

9 $$y_{1} = y_{0} + \varepsilon (y_{c} )_{0} = \frac{{c_{1} \varepsilon }}{2}e^{x/2}$$

and imposing the right hand side boundary condition yields

10 $$y_{1} = e^{(x - 1)/2}$$

Progressing in a similar way, the second iteration result is

11 $$y_{2} = e^{(x - 1)/2} + \frac{\varepsilon }{8}(1 - x)e^{(x - 1)/2}$$

This result can also be found with the method of matched asymptotic expansions. However, with PIA(1,1), the same solution is retrieved from a very simple initial guess. This solution does not satisfy the boundary condition at the left hand side i.e. $y_{2} (0) \ne 0$.

### The inner solution

The inner solution is valid within a small region called the boundary layer where the solution has a sharp turn. The first step is to determine the boundary layer variable by stretching the coordinate inside this layer

12 $$\xi = \frac{x}{{\varepsilon^{\nu } }}$$

Substituting the transformed variable into the original equation yields

13 $$\varepsilon^{1 - 2\nu } \frac{{d^{2} Y}}{{d\xi^{2} }} + 2\varepsilon^{ - \nu } \frac{dY}{d\xi } - Y = 0\quad \quad$$

where $Y = Y(\xi )$. Balancing should be performed for the terms. There are two options: Balancing the first and second term or the first and third term. Balancing the second and third term is not an option because *ν* = 0 and the original equation is retrieved with no stretching.

1. First and second term

For this case $1 - 2\nu = - \nu \; \Rightarrow \;\nu = 1$. Equation (13) after multiplication with *ε* is

14 $$\frac{{d^{2} Y}}{{d\xi^{2} }} + 2\frac{dY}{d\xi } - \varepsilon Y = 0$$

The goal is to retain as much terms as one can at the leading order. In this case, two terms remain in the first order and the choice is admissible.

2. First and third term

Balancing the first and third term requires $1 - 2\nu = 0\; \Rightarrow \;\nu = 1/2$. Equation (13) is

15 $$\frac{{d^{2} Y}}{{d\xi^{2} }} + 2\varepsilon^{ - 1/2} \frac{dY}{d\xi } - Y = 0$$

Since the middle term is the only leading dominant term, this choice is not an admissible choice and hence discarded.

To summarize, the distinguished limit is *ν* = 1 and the boundary layer variable is

16 $$\xi = \frac{x}{\varepsilon }$$

and the transformed equation to be solved is

17 $$F(Y,Y^{{\prime }} ,Y^{{\prime \prime }} ,\varepsilon ) = Y^{{\prime \prime }} + 2Y^{{\prime }} - \varepsilon Y = 0$$

Equation (3) takes the special form then

18 $$Y_{n}^{{\prime \prime }} + 2Y_{n}^{{\prime }} + 2\varepsilon (Y_{c}^{{\prime }} )_{n} + \varepsilon (Y_{c}^{{\prime \prime }} )_{n} - \varepsilon Y_{n} = 0,\quad n = 0,1,2, \ldots$$

Using (18) and (2) and applying the boundary condition at the left, i.e. *Y*(0) = 0, an iteration process can be constructed. Starting from a very simple guess

19 $$Y_{0} = 0$$

The successive two iteration are

20 $$Y_{1} = \varepsilon c_{3} (1 - e^{ - 2\xi } )$$

21 $$Y_{2} = \varepsilon c_{3} (1 - e^{ - 2\xi } ) + \varepsilon \left\{ {c_{7} (1 - e^{ - 2\xi } ) + \frac{{\varepsilon c_{3} }}{2}\xi \left( {1 + e^{ - 2\xi } } \right)} \right\}$$

Note that this solution is valid in the neighborhood of *x* = 0 and therefore is not expected to satisfy the boundary condition at the right hand side. The equations solved are second order while the conditions are less than the required value of 2. The undetermined coefficients will be determined by the matching conditions.

### Matching

The inner and outer solutions should smoothly combine in the overlapping region which requires solutions to be matched. Employing the Van Dyke’s (1975) matching principle

22 $$(y_{2} )^{i} = (Y{}_{2})^{0}$$

in the overlapping region. In accordance, the outer expansion is written in terms of the inner variable, approximated and equated to the inner expansion written in terms of the outer variable and approximated. Hence

23 $$(y_{2} )^{i} = e^{(\varepsilon \xi - 1)/2} + \frac{\varepsilon }{8}(1 - \varepsilon \xi )e^{(\varepsilon \xi - 1)/2}$$

and then approximation is taken up to two terms for fixed *ξ*

24 $$(y_{2} )^{i} \cong e^{ - 1/2} \left( {1 + \varepsilon \frac{\xi }{2}} \right) + \frac{\varepsilon }{8}e^{ - 1/2} + \ldots$$

Returning back to the original variable *x* now

25 $$(y_{2} )^{i} \cong e^{ - 1/2} \left( {1 + \frac{x}{2}} \right) + \frac{\varepsilon }{8}e^{ - 1/2} + \ldots$$

For the right hand side of the equation in the matching, the inner expansion is represented in terms of the outer variable *x*

26 $$(Y_{2} )^{0} = \varepsilon c_{3} (1 - e^{ - 2x/\varepsilon } ) + \varepsilon \left( {c_{7} (1 - e^{ - 2x/\varepsilon } ) + \frac{{\varepsilon c_{3} }}{2}\frac{x}{\varepsilon }\left( {1 + e^{ - 2x/\varepsilon } } \right)} \right)$$

and approximated up to two terms for fixed *x*

27 $$(Y_{2} )^{0} \cong \varepsilon c_{3} (1 + \frac{x}{2}) + \varepsilon c_{7}$$

Note that for *x* kept fixed and not negligibly small, $e^{ - 2x/\varepsilon }$ is an exponentially small term which can be neglected. Equating (27) and (25), the constants

28 $$\varepsilon c_{3} = e^{ - 1/2} ,\quad c_{7} = \frac{1}{8}e^{ - 1/2}$$

are determined from the matching conditions. Hence the final inner and outer solutions in terms of the original spatial variable are

29 $$Y_{2} = e^{ - 1/2} (1 - e^{ - 2x/\varepsilon } ) + \frac{1}{2}e^{ - 1/2} x(1 + e^{ - 2x/\varepsilon } ) + \frac{\varepsilon }{8}\,e^{ - 1/2} \left( {1 - e^{ - 2x/\varepsilon } } \right)$$

30 $$y_{2} = e^{(x - 1)/2} + \frac{\varepsilon }{8}(1 - x)e^{(x - 1)/2}$$

The inner solution is valid in the neighborhood of *x* = 0 (inside the boundary layer) and the outer solution is valid outside the boundary layer. A solution which is valid throughout the domain is desirable and one can construct a composite solution valid within all the domain of interest

31 $$y = Y_{2} + y_{2} - (y_{2} )^{i}$$

To construct the composite expansion, one simply adds the inner and outer solutions and subtracts the common overlapping part which is counted twice from the solution. Substituting (29), (30) and (25) into (31), the composite expansion is

32 $$y = e^{(x - 1)/2} - \left( {1 - \frac{x}{2}} \right)e^{ - 1/2 - 2x/\varepsilon } + \frac{\varepsilon }{8}\left\{ {(1 - x)e^{(x - 1)/2} - e^{ - 1/2 - 2x/\varepsilon } } \right\}\,$$

which is a valid approximation throughout the whole domain. This solution satisfies both of the boundary conditions and can also be retrieved by the method of matched asymptotic expansions. To compare with the exact solution of the problem

33 $$y = \frac{{e^{{\left( { - 1 + \sqrt {1 + \varepsilon } } \right)x/\varepsilon }} - e^{{\left( { - 1 - \sqrt {1 + \varepsilon } } \right)x/\varepsilon }} }}{{e^{{\left( { - 1 + \sqrt {1 + \varepsilon } } \right)/\varepsilon }} - e^{{\left( { - 1 - \sqrt {1 + \varepsilon } } \right)/\varepsilon }} }}$$

first, the Taylor series expansions are written

34 $$\sqrt {1 + \varepsilon } \cong 1 + \frac{1}{2}\varepsilon - \frac{1}{8}\varepsilon^{2}$$

35 $$e^{{( - 1 + \sqrt {1 + \varepsilon } )/\varepsilon }} \cong e^{1/2 - \varepsilon /8}$$

36 $$e^{{( - 1 - \sqrt {1 + \varepsilon } )/\varepsilon }} \cong e^{ - 2/\varepsilon - 1/2 + \varepsilon /8} \cong 0$$

The second term is an exponentially small term which can be neglected. Under the approximations, the exact solution is

37 $$y^{e} \cong e^{ - 1/2 + \varepsilon /8} e^{x/2 - \varepsilon x/8} - e^{ - 1/2 + \varepsilon /8} e^{ - 2x/\varepsilon - x/2 + \varepsilon x/8}$$

Approximating further the exponential terms with the perturbation parameter

38 $$e^{\varepsilon /8} \cong 1 + \frac{\varepsilon }{8}$$

39 $$e^{ - \varepsilon x/8} \cong 1 - \frac{\varepsilon }{8}x$$

the result is

40 $$y \cong e^{ - 1/2 + x/2} - e^{ - 1/2 - 2x/\varepsilon - x/2} + \frac{\varepsilon }{8}\left\{ {(1 - x)e^{ - 1/2 + x/2} - (1 + x)e^{ - 1/2 - 2x/\varepsilon - x/2} } \right\}$$

This approximation is comparable with the perturbation iteration solution if further

41 $$e^{ - x/2} \cong 1 - \frac{x}{2}$$

is taken in (40).

### Example Problem 2

Consider the non-homogenous linear boundary value problem

42 $$\varepsilon y^{{\prime \prime }} + 2y^{{\prime }} = x\quad y(0) = \alpha ,\quad y(1) = \beta$$

### The outer solution

To find the outer solution, the original equation is used

43 $$F(y^{{\prime }} ,y^{{\prime \prime }} ,\varepsilon ) = \varepsilon y^{{\prime \prime }} + 2y^{{\prime }} - x = 0$$

The boundary layer is located near *x* ≈ 0. Therefore, the outer solution is not expected to satisfy the condition at *x* = 0. Substituting (43) into (3) and rearranging, the iteration equation is

44 $$2y_{n}^{{\prime }} - x + 2\varepsilon (y_{c}^{{\prime }} )_{n} + \varepsilon y_{n}^{{\prime \prime }} = 0,\quad n = 0,1,2, \ldots$$

Choose a simple initial guess compatible with the boundary condition at the right

45 $$y_{0} = \beta$$

to start the iteration. The first iteration solution satisfying the boundary condition at the right is

46 $$y_{1} = \beta + \frac{{x^{2} - 1}}{4}$$

This solution is not expected to satisfy the boundary condition at the left hand side i.e. $y_{1} (0) \ne 0$.

### The inner solution

The inner solution is valid within a small region called the boundary layer where the solution has a sharp turn. It turns out that the boundary layer variable by stretching the coordinate inside this layer is

47 $$\xi = \frac{x}{\varepsilon }$$

and the transformed equation is

48 $$\frac{{d^{2} Y}}{{d\xi^{2} }} + 2\frac{dY}{d\xi } - \varepsilon^{2} \xi = 0$$

Defining

49 $$F(Y^{{\prime }} ,Y^{{\prime \prime }} ,\varepsilon ) = Y^{{\prime \prime }} + 2Y^{{\prime }} - \varepsilon^{2} \xi = 0$$

the iteration Eq. (3) takes the form

50 $$Y_{n}^{{\prime \prime }} + 2Y_{n}^{{\prime }} + 2\varepsilon (Y_{c}^{{\prime }} )_{n} + \varepsilon (Y_{c}^{{\prime \prime }} )_{n} = 0,\quad n = 0,1,2, \ldots$$

Starting from a simple guess satisfying the boundary condition at the left

51 $$Y_{0} = \alpha$$

the first iteration is

52 $$Y_{1} = \alpha + \varepsilon c_{3} (1 - e^{ - 2\xi } )$$

Note that this solution is valid in the neighborhood of *x* = 0 and therefore is not expected to satisfy the boundary condition at the right hand side. The undetermined coefficient will be determined by the matching conditions.

### Matching

The matching of solutions requires

53 $$(y_{1} )^{i} = (Y{}_{1})^{0}$$

in the overlapping region. In accordance, the outer expansion is written in terms of the inner variable, approximated and equated to the inner expansion written in terms of the outer variable and approximated. Hence

54 $$(y_{1} )^{i} = \beta + \frac{{\varepsilon^{2} \xi^{2} - 1}}{4} \cong \beta - \frac{1}{4}$$

The inner expansion is represented in terms of the outer variable *x* and approximated

55 $$(Y_{1} )^{0} = \alpha + \varepsilon c_{3} (1 - e^{ - 2x/\varepsilon } ) \cong \alpha + \varepsilon c_{3}$$

Note that for *x* kept fixed and not negligibly small, $e^{ - 2x/\varepsilon }$ is an exponentially small term which can be neglected. Equating (54) and (55) gives the undetermined constant

56 $$\varepsilon c_{3} = \beta - \frac{1}{4} - \alpha$$

Hence the inner solution in terms of the original variable is

57 $$Y_{1} = \beta - \frac{1}{4} + \left( {\alpha - \beta + \frac{1}{4}} \right)e^{ - 2x/\varepsilon }$$

The composite solution is

58 $$y = Y_{1} + y_{1} - (y_{1} )^{i}$$

or

59 $$y = \beta + \frac{{x^{2} - 1}}{4} + \left( {\alpha - \beta + \frac{1}{4}} \right)e^{ - 2x/\varepsilon }$$

which is a valid approximation throughout the whole domain. This solution satisfies both of the boundary conditions and can also be retrieved by the method of matched asymptotic expansions.The exact solution of the problem is

60 $$y = \alpha + \frac{{\alpha - \beta + \frac{1}{4} - \frac{1}{4}\varepsilon }}{{1 - e^{ - 2/\varepsilon } }}\left( {e^{ - 2x/\varepsilon } - 1} \right) + \frac{1}{4}x^{2} - \frac{1}{4}\varepsilon x$$

Neglecting the exponentially small term $e^{ - 2/\varepsilon }$ and *O*(*ε*) terms, solution (59) is obtained, hence the solution is a valid approximation of the exact solution.

## Applications to non-linear differential equations

A first order and a second order nonlinear boundary value problems are solved in this section using the method.

### Example Problem 3

Consider the first order non-linear boundary value problem

61 $$\varepsilon y^{{\prime }} + y^{2} = 4\quad y(0) = 1$$

### The outer solution

For the outer solution, the original equation is used

62 $$F(y,y^{{\prime }} ,\varepsilon ) = \varepsilon y^{{\prime }} + y^{2} - 4 = 0$$

The boundary layer is assumed to be located near $x \approx 0$. If there arises any inconsistency in the solutions, the assumption may be revised. The outer solution is not expected to satisfy the condition at *x* = 0. Substituting (62) into (3) and re-arranging, the iteration equation is

63 $$y_{n}^{2} - 4 + 2y_{n} \varepsilon (y_{c} )_{n} + \varepsilon y_{n}^{{\prime }} = 0,\quad n = 0,1,2, \ldots$$

Since there is no boundary condition at the right hand side, the initial guess might be selected so that the iteration equation takes the simplest form. Hence

64 $$y_{0} = 2$$

The first iteration solution is

65 $$y_{1} = 2$$

which is the same solution with the starting value. Therefore the outer expansion seems to be converged.

### The inner solution

The boundary layer variable by stretching the coordinate inside this layer is

66 $$\xi = \frac{x}{\varepsilon }$$

and the transformed equation is

67 $$\frac{dY}{d\xi } + Y^{2} - 4 = 0$$

The iteration Eq. (3) takes the form

68 $$Y_{n}^{\prime } + Y_{n}^{2} - 4 + 2Y_{n} \varepsilon (Y_{c} )_{n} + \varepsilon (Y_{c}^{\prime } )_{n} = 0,\quad n = 0,1,2, \ldots$$

Starting from a simple guess

69 $$Y_{0} = 2$$

which makes the most simplification in the iteration equation, the successive iterations are

70 $$Y_{1} = 2 - e^{ - 4\xi }$$

71 $$Y_{2} = 2 - \frac{5}{4}e^{ - 4\xi } + \frac{1}{4}e^{ - 8\xi }$$

### Matching

The matching of solutions requires

72 $$(y_{2} )^{i} = (Y{}_{2})^{0}$$

in the overlapping region. Since the outer solution is constant, the expansion and approximation in the inner variable is the same

73 $$(y_{2} )^{i} = 2$$

The inner expansion is represented in terms of the outer variable *x* and approximated

74 $$(Y_{2} )^{0} = 2 - \frac{5}{4}e^{ - 4x/\varepsilon } + \frac{1}{4}e^{ - 8x/\varepsilon } \cong 2$$

The solutions are in agreement in the overlapping region. The composite expansion valid throughout the domain is

75 $$y = 2 - \frac{5}{4}e^{ - 4x/\varepsilon } + \frac{1}{4}e^{ - 8x/\varepsilon }$$

The exact solution is

76 $$y = 2\tanh \left[ {\tanh^{ - 1} \left( {\frac{1}{2}} \right) + \frac{2x}{\varepsilon }} \right]$$

**Fig. 1:**
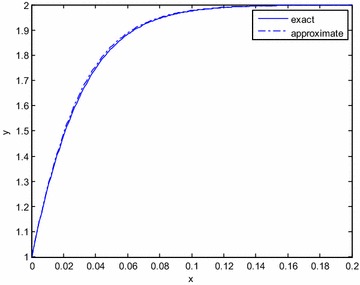
Comparison of the exact and approximate solutions for *ε* = 0.1 (Example Problem 3)

**Fig. 2:**
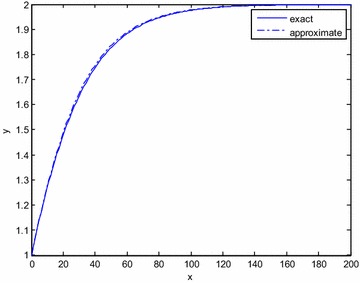
Comparison of the exact and approximate solutions for *ε* = 100 (Example Problem 3)

Solution (75) is a very good approximation of the exact solution (76). In Fig. 1, *ε* = 0.1, and in Fig. 2, *ε* = 100 are taken. For small or large perturbation parameters, the agreement is excellent.

### Example Problem 4

Consider the second order non-linear boundary value problem (Cole 1968)

77 $$\varepsilon y^{{\prime \prime }} + yy^{{\prime }} - y = 0\quad y(0) = 0,\quad y(1) = 3$$

### The outer solution

For the outer solution, the original equation is used

78 $$F(y,y^{{\prime }} ,y^{{\prime \prime }} ,\varepsilon ) = \varepsilon y^{{\prime \prime }} + yy^{{\prime }} - y = 0$$

The boundary layer is located near *x* ≈ 0. The outer solution is not expected to satisfy the condition at *x* = 0. Substituting (78) into (3) and rearranging, the iteration equation is

79 $$y_{n} y_{n}^{{\prime }} - y_{n} + (y_{n}^{{\prime }} - 1)\varepsilon (y_{c} )_{n} + y_{n} \varepsilon (y_{c}^{{\prime }} )_{n} + \varepsilon y_{n}^{{\prime \prime }} = 0,\quad n = 0,1,2, \ldots$$

*y*_0_ = 3 which is inspired from the right hand side boundary condition would not be a good initial guess. Rather, one has to look for a trivial function which most simplifies the iteration equation for *n* = 0. Such an initial guess is

80 $$y_{0} = x$$

The first and second iteration solutions satisfying the right hand side condition is

81 $$y_{1} = x + 2$$

82 $$y_{2} = x + 2$$

which shows that the outer solution converged.

### The inner solution

The boundary layer variable by stretching the coordinate inside this layer is

83 $$\xi = \frac{x}{\varepsilon }$$

and the transformed equation is

84 $$\frac{{d^{2} Y}}{{d\xi^{2} }} + Y\frac{dY}{d\xi } - \varepsilon Y = 0$$

The iteration Eq. (3) takes the form

85 $$Y_{n}^{{\prime \prime }} + Y_{n} Y_{n}^{{\prime }} + Y_{n}^{{\prime }} \varepsilon (Y_{c} )_{n} + Y_{n} \varepsilon (Y_{c}^{{\prime }} )_{n} + \varepsilon (Y_{c}^{{\prime \prime }} )_{n} - \varepsilon Y_{n} = 0,\quad n = 0,1,2, \ldots$$

Starting from a simple guess

86 $$Y_{0} = 2$$

which makes the most simplification in the iteration equation, the first iteration is

87 $$Y_{1} = (2 + c_{1} )(1 - e^{ - 2\xi } ) + \varepsilon \xi$$

Matching this solution with the first iteration of the outer solution makes a simplification and the constant *c*_1_ turns out to be zero. Hence, the final first iteration is

88 $$Y_{1} = 2(1 - e^{ - 2\xi } ) + \varepsilon \xi$$

The second iteration yields

89 $$Y_{2} = 2\left( {1 - e^{ - 2\xi } } \right) + \varepsilon \xi \left( {1 + e^{ - 2\xi } } \right) + e^{ - 4\xi } + \varepsilon c_{2} + \left( {\varepsilon \xi^{2} - 1 - \varepsilon c_{2} } \right)e^{ - 2\xi }$$

### Matching

The matching of solutions requires

90 $$(y_{2} )^{i} = (Y{}_{2})^{0}$$

in the overlapping region. The outer solution is written in the inner variable and approximated including first order terms yield the same result

91 $$(y_{2} )^{i} = 2 + \varepsilon \xi = 2 + x$$

Then the inner expansion is represented in terms of the outer variable *x* and approximated

92 $$(Y_{2} )^{0} = 2\left( {1 - e^{ - 2x/\varepsilon } } \right) + x\left( {1 + e^{ - 2x/\varepsilon } } \right) + e^{ - 4x/\varepsilon } + \varepsilon c_{2} + \left( {\frac{{x^{2} }}{\varepsilon } - 1 - \varepsilon c_{2} } \right)e^{ - 2x/\varepsilon } \cong 2 + x + \varepsilon c_{2}$$

since terms of $e^{ - 2x/\varepsilon } ,\;e^{ - 4x/\varepsilon }$ are exponentially small in the overlapping region. Comparing (92) with (91), it is found that *c*_2_ = 0. The inner expansion in the final form is

93 $$Y_{2} = 2\left( {1 - e^{ - 2\xi } } \right) + \varepsilon \xi \left( {1 + e^{ - 2\xi } } \right) + e^{ - 4\xi } + \left( {\varepsilon \xi^{2} - 1} \right)e^{ - 2\xi }$$

The composite expansion is calculated as a final step

94 $$y = Y_{2} + y_{2} - (y_{2} )^{i}$$

or

95 $$y = 2 - 3e^{ - 2x/\varepsilon } + x\left( {1 + e^{ - 2x/\varepsilon } } \right) + e^{ - 4x/\varepsilon } + \frac{{x^{2} }}{\varepsilon }e^{ - 2x/\varepsilon }$$

**Fig. 3:**
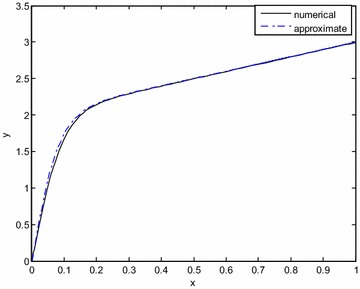
Comparison of the exact and approximate solutions for *ε* = 0.1 (Example Problem 4)

**Fig. 4:**
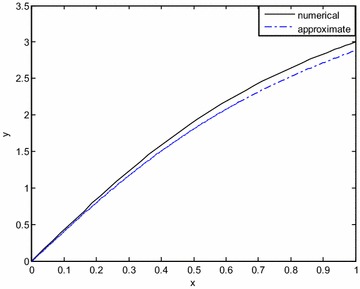
Comparison of the exact and approximate solutions for *ε* = 1 (Example Problem 4)

Solution (95) is contrasted with the numerical solution of the original equation obtained by an adaptive step size Runge–Kutta method combined with shooting. In Fig. 3, *ε* = 0.1 is taken and the match is excellent. In Fig. 4, a larger perturbation parameter, namely *ε* = 1 is taken and there is a reasonably good agreement between the results.

## Concluding remarks

Boundary layer type problems are treated by the perturbation iteration method for the first time. The simplest algorithm of PIA(1,1) in which there is only one correction term in the perturbation expansion and at most first order derivatives in the Taylor series expansions is considered. For PIA(1,1), due to the reduction of differential order of the iterated equations, an outer and an inner expansion must be found independently via the appropriate iteration procedures. The solutions are then matched with each other to construct a composite expansion valid throughout the whole domain. Singular linear and nonlinear problems are treated with the method and results are compared with the available analytical or numerical solutions. It is shown that the method can be effectively applied to singular problems producing admissible analytical approximate solutions.

## References

[CR1] Aksoy Y, Pakdemirli M (2010). New perturbation–iteration solutions for Bratu-type equations. Comput Math Appl.

[CR2] Aksoy Y, Pakdemirli M, Abbasbandy S, Boyacı H (2012). New perturbation–iteration solutions for nonlinear heat transfer equations. Int J Numer Methods Heat Fluid Flow.

[CR3] Bluman GW, Kumei S (1989). Symmetries and differential equations.

[CR4] Cole JD (1968). Perturbation methods in applied mathematics.

[CR5] Dolapci IT, Şenol M, Pakdemirli M (2013). New perturbation iteration solutions for Fredholm and Volterra integral equations. J Appl Math.

[CR6] He JH (2001). Iteration perturbation method for strongly nonlinear oscillators. J Vib Control.

[CR7] Mickens RE (1987). Iteration procedure for determining approximate solutions to nonlinear oscillator equations. J Sound Vib.

[CR8] Mickens RE (2005). A generalized iteration procedure for calculating approximations to periodic solutions of ‘‘Truly Nonlinear Oscillators’’. J Sound Vib.

[CR9] Mickens RE (2006). Iteration method solutions for conservative and limit-cycle force oscillators. J Sound Vib.

[CR10] Nayfeh AH (1981). Introduction to perturbation techniques.

[CR11] Pakdemirli M (2013). Review of the new perturbation iteration method. Math Comput Appl.

[CR12] Pakdemirli M (2015). Perturbation–iteration method for strongly nonlinear vibrations. J Vib Control.

[CR13] Pakdemirli M, Boyacı H (2007). Generation of root finding algorithms via perturbation theory and some formulas. Appl Math Comput.

[CR14] Pakdemirli M, Boyacı H, Yurtsever HA (2007). Perturbative derivation and comparisons of root-finding algorithms with fourth order derivatives. Math Comput Appl.

[CR15] Pakdemirli M, Boyacı H, Yurtsever HA (2008). A root finding algorithm with fifth order derivatives. Math Comput Appl.

[CR16] Pakdemirli M, Aksoy Y, Boyacı H (2011). A new perturbation–iteration approach for first order differential equations. Math Comput Appl.

[CR17] Şenol M, Dolapci IT, Aksoy Y, Pakdemirli M (2013). Perturbation–iteration method for first-order differential equations and systems. Abstr Appl Anal.

[CR18] Van Dyke M (1975). Perturbation methods in fluid mechanics.

